# Medical Items

**Published:** 1898-04

**Authors:** 


### *MEDICAL ITEMS.*

MATHEWS *Quarterly Journal of Rectal Gastro-Intestinal Diseases* will hereafter appear as a monthly under the name of the *Louisville Journal of Surgery and Medicine.*

TO STOP an epistaxis, it is suggested in an article before us to insert a rubber condom well back in the nares and fill it up with finely cracked ice. *Secondom artem,* isn't it?

WHAT'S the matter with Keokuk, Iowa? Under the "Change of Address" heading in the last *Journal of the American Medical Association,* we notice that ten doctors have just left that town for other more promising fields. As a good place to get away from Keokuk seems to be quite a success.

IT is reported that over forty victims of the cocain babit, induced largely by the use of popular preparations as catarrh cures, appeared in the police courts, etc., of Chicago within two or three months of last year. The evil was so noticeable as to cause the introduction of an ordinance prohibiting the sale of these dangerous remedies.—*Ex.*

DR. A. G. HOBBS has removed his office from 14½ Whitehall street to elegant new offices in the English-American Trust Co.'s building on Peachtree street. Dr. Hobbs was in his old offices for sixteen years, and we trust that he will enjoy the same good health and prosperity, now that he has moved to a more modern building. He is beautifully fitted up on the seventh floor.

THE reorganization of the old Savannah Medical College (which has been dead for thirty years) is being discussed. What imaginary reasons any persons can have for creating another medical college is past our understanding. There is no need for a new college in Savannah or anywhere else in this country. The oversupply which we already have is one of the evils which afflict the medical profession to-day, and to those who contemplate adding still further to this excess we sincerely urge, *Don't*, for heaven's sake, DON'T.

---

MENTION was made in our last issue of Dr. J. J. Devine, of Texas, as one of our oldest subscribers. Probably the oldest on our books is Dr. T. J. Mitchell, of Locust Grove, Georgia. Dr. Mitchell first became a subscriber in 1859, and when the JOURNAL was revived after the war he again subscribed, and through all the varying fortunes of the JOURNAL since that date he has not forgotten us. Dr. Mitchell has had a long and useful medical experience, and we would be pleased if he would give us some of his professional reminiscences for publication.

---

AN important innovation in the plan of Sajous' *Annual of the Universal Medical Sciences* is announced by the editor. Beginning with 1898 a new classification and arrangement, more adapted to the use of the general practitioner and yet of no less value to the medical author, will be adopted. Instead of presenting the excerpts from the year's literature arranged in order under a general head as before, each disease is described *in extenso* and the new features of the past year properly noted. The work therefore becomes a complete reference text-book of medical science revised each year. The volumes are awaited with interest, as the new plan promises largely increased usefulness.—*Ex.*

THE New York Skin and Cancer Hospital has moved out of its old quarters on Thirty-fourth street into its handsome new building on Nineteenth street and Second avenue. The new building is a modern fire-proof structure of four stories and basement, containing about sixty beds for patients; of these seven are in private rooms. On the top floor is a modern operating-room, well equipped, the Worden ward for children, and most of the private rooms. On the next two floors are four wards for male and female patients with diseases of the skin and cancer. On the ground floor is the large out-patient room, with consulting rooms, pathological laboratory, drug room, office, reception room, etc. In the basement there is a complete set of baths, including Turkish, Russian, needle, and plunge, besides the kitchen, laundry, dining-room, etc. The lighting, heating and ventilation are of the most approved kind.

---

THE late death of Dr. Hugh Hagan was indeed a grievous loss to the profession of this city, and no one has gone to his grave whose absence will be more deeply felt by surviving friends. Dr. Hagan was born in Richmond, Virginia, in 1863. He graduated in Medicine at the College of Physicians and Surgeons, New York, in 1888, and after a year's study abroad he began to practice his profession in Atlanta. In his professional work he was eminently successful. His rise was rapid, and by innate merit and an agreeable personality he steadily acquired a large and lucrative practice. He was a constant student of medicine, and when not occupied with the demands of his busy professional life, he could usually be found in his library among his books and journals. As a physician he was scientific but conservative; he was patient and accurate; he possessed a keen and discriminating judgment in diagnosis, and he was careful and faithful in the management and treatment of his patients. Dr. Hagan was in the prime of an apparently vigorous manhood, and his sudden death from apoplexy will be sincerely and genuinely mourned by a host of friends, lay and professional. As a man he possessed the noble traits of a high personal character; he was kind, generous, conscientious, honest, unassuming, and warm-hearted, and he wore "without abuse the grand old name of gentleman." "He should have died hereafter."

---

THE third annual meeting of the Association of Surgeons of the Southern Railway Company will convene at the Hygeia Hotel, Old Point Comfort, Virginia, Tuesday and Wednesday, June 21 and 22, 1898.

At the same time and place it is proposed to hold a conference of chief surgeons or other medical representatives of the principal railroads operating in Virginia, West Virginia, North and South Carolina, Georgia, Florida, Alabama, Mississippi, Louisiana, Texas, Arkansas, Tennessee, and Kentucky, for the purpose of determining the advisability of forming an organization to be known as the "Southern Association of Railway Surgeons," or some other equally comprehensive name. The following distinguished surgeons have promised to be present and to deliver addresses on subjects pertaining to railway surgery: Dr John A. Wyeth, New York; Drs. W. W. Keen and Joseph Price, Philadelphia; Dr. Hunter McGuire, Richmond; Dr. Walter Wyman, Surgeon-General U. S. Marine Hospital Service; Dr. Joseph Ransohoff, Cincinnati; Dr. Willis F. Westmoreland, Atlanta, and Dr. J. J. Kinyoun, Past Assistant Surgeon, U. S. Marine Hospital Service. (C. M. Drake, M.D., President; T. H. Hancock, M.D., Secretary, 6½ Whitehall St., Atlanta, Ga.)

